# An ideal solution? Optimising pretreatment methods for artificially mummified ancient Egyptian tissues

**DOI:** 10.1002/rcm.8686

**Published:** 2020-02-19

**Authors:** Jenefer Cockitt, Angela Lamb, Ryan Metcalfe

**Affiliations:** ^1^ KNH Centre for Biomedical Egyptology University of Manchester Oxford Road Manchester M13 9PL UK; ^2^ National Environmental Isotope Facility British Geological Survey Keyworth, Nottingham NG12 5GG UK

## Abstract

**Rationale:**

Although the analysis of skeletal remains dominates the study of ancient dietary stable isotopes, mummified bodies also allow short‐term diet to be studied through the analysis of soft tissues. The application of resins, waxes and oils during mummification can affect the results obtained. This study assesses a range of methods for removing such substances from mummified tissue.

**Methods:**

An experimental mummification model following ancient Egyptian methods was created using a modern pig leg. Sub‐samples of skin, muscle and bone were removed and coated with a range of substances used in Egyptian mummification. Four methods were used to clean these samples before the measurement of the carbon and nitrogen stable isotope ratios of their gelatinised collagen content using a ThermoFinnigan Flash Elemental analyser coupled to a DeltaPlus XL isotope ratio mass spectrometer via a ConFlo III interface.

**Results:**

The results showed that embalming materials can significantly affect dietary stable isotope ratios, and that these substances are most effectively removed using a mixture of polar and non‐polar solvents. Results indicate that bone samples demineralised with HCl and skin samples produce more accurate results than bone samples demineralised with EDTA or muscle samples.

**Conclusions:**

The choice of tissue and the preparation methods used can have a significant effect on the accuracy of stable isotope data obtained from mummified tissue, particularly when embalming materials are also present. A mixture of solvents appears to be a more effective cleaning agent than a single solvent. Demineralisation with HCl is preferable for well‐preserved bone, as used in this study, but whether this is the case for more fragile, less well‐preserved bone requires further study. Skin samples produce more consistent data than muscle, but visually distinguishing between these tissues is not simple on ancient mummies.

## 1 INTRODUCTION

The application of dietary stable isotope analysis to mummified human remains from ancient Egypt is an area of research that remains largely unexplored.^1^ Despite the palaeopathological interest in mummies there have been few studies that use the preserved tissues for the measurement of stable isotopes. Although there are a number of published works looking at carbon, nitrogen, oxygen and sulphur isotope ratios from ancient Egyptian human remains,^2^, ^3^, ^4^ the majority of these use bone samples taken from skeletal remains that were not subject to the complex artificial mummification process employed in ancient Egypt throughout most of the pharaonic period. The range of tissues frequently preserved in mummies (bone, tooth, skin, muscle, hair and nail) makes them a valuable resource for the study of both long‐ and short‐term dietary isotopes that is not currently being used to its full potential.

The survival of mummified human remains in ancient Egypt is due to two factors: (1) the arid climate of the region, and (2) the range of physical and chemical techniques employed by the ancient Egyptians to artificially preserve their dead. There are a significant number of bodies from the pharaonic period and later that demonstrate varying degrees of preservation due to the simple placement of the body in the hot sand after death. Similar instances of natural mummification are also known from neighbouring Nubia, where preserved soft tissues and hair have been subject to some stable isotope analyses.^5^, ^6^ It is, however, the second method of preservation which has produced the large number of mummified bodies for which Egypt is well known.^7^ Although the anthropogenic mummification process used by embalmers has been well studied, the implications of this process for a range of different methods of modern scientific analysis have not. This paper attempts to address this question with regard to carbon and nitrogen stable isotope analysis and to present some suggestions for ensuring future production of reliable analyses of mummified tissues.

### 1.1 Ancient Egyptian mummification

The methods used to artificially preserve the body in ancient Egypt are well understood.^8^, ^9^, ^10^ The main features of mummification were the removal of the internal organs, desiccation of the body using natron (a naturally occurring mixture of sodium salts found in dried lake beds) and the application of various unguents (salves) and resins. Some of these resins are thought to have aided the mummification process by prohibiting the action of bacteria and/or by creating a physical barrier over the mummies' skin and internal cavities to prevent insect ingress.^11^ Others such as myrrh were perfumed and are likely to have helped mask the smell of decay. The application of resinous materials to a mummified body is known to vary according to the quality of the embalming method employed and the historical age of the mummy.^7^

Considerable analysis has been conducted to identify the constituents of embalming resins and their geographical origins using gas chromatography linked to mass spectrometry (GC/MS).^12^, ^13^, ^14^, ^15^ As a result, several key resins and waxes have been identified as recurring throughout the periods when resin application was favoured (from the 18^th^ Dynasty onwards). These include beeswax, animal fats, pine resin and bitumen (often from the Dead Sea area^13^), alongside more infrequently used substances such as pistacia resin and myrrh.^14^ Many of the coatings found on mummified bodies have also been identified as mixtures of a series of these substances.

As the majority of ancient Egyptian mummies available for analysis come from the later periods of Egyptian history when the use of various resins was more common, it is important to determine whether these resins can be adequately removed from a sample prior to stable isotope analysis. As residual resin within a sample is organic in origin it has the potential to contaminate the stable isotope signature.

### 1.2 The implications of artificial mummification for stable isotope analysis

The presence of resin within a soft tissue or bone sample has previously caused issues, in particular with material submitted for radiocarbon analysis. Both pine resin and bitumen have inherent radiocarbon ages and so must be removed prior to dating. The acid–base–acid pretreatment methods used commonly in AMS (Accelerator Mass Spectrometry) radiocarbon dating have been found not to successfully remove these contaminants, resulting in the production of anomalous radiocarbon ages.^16^ A study from the University of Minnesota^17^ did, however, demonstrate that benzene would successfully remove resin from samples submitted for radiocarbon analysis without affecting the radiocarbon age or stable isotope ratios of ancient material (liver, muscle, hair and linen). One of the two published stable isotope papers dealing with artificially mummified remains also reports attempts to remove contaminating resin, in this case from ancient Egyptian soft tissue and hair.^18^ Different cleaning methods, based largely on sequences of chloroform and methanol, were applied to the samples with success judged by the degree of change in the δ^13^C values. In the absence of true baseline values for the ancient tissues, however, it is impossible to fully determine the success of these methods.

### 1.3 Wider significance

The ability to reliably remove embalming materials from soft tissue is of fundamental importance for palaeodietary research. Bone samples are predominantly used in palaeodietary stable isotope analysis due to the preponderance of skeletal remains in the archaeological record compared with mummified remains. Using a combination of different tissue types, where available, can offer significantly greater insights into the diet of an individual or population, as the different tissue turnover rates allow dietary inference over different time periods. Analysis of collagen from bone can provide information on diet over decades, whereas collagen from skin and muscle can provide information of the order of weeks to months.^19^ Analysis of hair in 1–2 cm sections can provide information on a similar scale to muscle collagen but, by analysing sections from root to tip, it is also possible to gain insight into the seasonality of diet.^20^ As such, the development of a successful preparatory method for mummified Egyptian tissues will potentially enlarge the array of material suitable for analysis and, in doing so, will greatly expand understanding of ancient Egyptian diet, health and lifestyle.

Despite being perhaps most commonly associated with ancient Egyptian mummies, the application of animal‐ and plant‐based coatings is also seen in several other cultures. Anthropogenically created mummies from Peru and Chile, the Canary Islands and Australia have all shown evidence for the use of such embalming materials,^21^, ^22^, ^23^ making them potentially subject to the same inherent pre‐treatment problems. A preparatory method that can remove the range of embalming substances used in Egyptian mummies would provide at least a starting point for the development of similar methods for mummies from these other cultures.

### 1.4 Aim

For this pilot study, our approach is based exclusively on the use of a modern animal model rather than ancient tissues. This allows for the assessment of a range of cleaning methods and the provision of untreated and non‐decomposed samples of skin, muscle and bone as controls that can be used to provide a reliable baseline for the interpretation of the other samples. Such a baseline cannot be provided for ancient tissues, as they are necessarily affected by a complex array of chemical changes caused by time, embalming methods, and degradation. Their use in preliminary testing of experimental methods such as that described herein is also inappropriate from an ethical standpoint.

Pig tissue was selected as the generally accepted closest analogue to human tissue and a leg from an organically reared pig was selected. Organically reared pigs in the UK have a diet similar to the ancient Egyptian diet, i.e., mainly C3 plants, with a small quantity of C4 plants and fish protein. The leg was artificially mummified using a synthetic natron mix and, once desiccated, sub‐samples were coated with various resins, oils and other substances, both alone and in mixtures, with uncoated tissues used as further controls. Hereon in, the different methods of preparing the tissues will be referred to as ‘embalming treatments’ and the different coatings used as ‘embalming materials’. Five pretreatments (detailed in section 2) were then used in an attempt to remove these substances using common stable isotope and radiocarbon pretreatment methods, as well as other methods based on these protocols. Hereon in, these pretreatment methods will be referred to as ‘cleaning methods’. We aim to identify a cleaning method that will remove contaminants effectively without causing undue damage to the tissue itself, or affect carbon or nitrogen stable isotope ratios.

## 2 MATERIALS AND METHODS

A full rear leg weighing 9.85 kg with skin and foot intact from a free‐range reared pig was obtained from a butcher. Samples of muscle, skin and bone were taken immediately to act as fresh tissue controls (embalming treatment no. 1) and stored at −80°C. As previous experiments have shown^24^, ^25^ that a ratio of around 5:1 (w/w) natron/tissue is sufficient for mummification, 50 kg of synthetic natron was prepared from 25 kg anhydrous sodium carbonate (Extra Pure grade; Sigma, Gillingham, UK), 15 kg sodium hydrogen carbonate (VWR RECTAPUR, Lutterworth, UK), 5 kg sodium sulphate (VWR RECTAPUR) and 5 kg sodium chloride (99.5+%; Fisher, Loughborough, UK). This ratio is similar to that of naturally occurring deposits of natron from Egypt that have been shown to effectively promote mummification in previous experiments.^24^ The leg was placed on a 5–7 cm thick layer of natron weighing approx. 15 kg, with the remaining approximately 35 kg covering it completely.

After 40 days, samples of skin, muscle and bone were taken from the lower leg, as the upper leg showed signs of significant decomposition. All samples were taken from sites immediately adjacent to each other to reduce the possible impact of sampling site on the stable isotope ratios obtained. These samples were further sub‐divided into four parts, each receiving a different embalming treatment as detailed in Table 1.

**Table 1:** Embalming treatments applied to mummified skin after 40 days. All percentage compositions are %w/w. Pine resin, beeswax and pistacia resin obtained from G. Baldwin & Co. (London, UK), palm oil from http://www.natr.co.uk

Embalming treatment no.	Method	Ref.
2	Brushed clean to act as mummification control	N/A
3	Brushed clean and coated with 100% pine resin	N/A
4	Brushed clean and coated with 37% pine resin, 35% palm oil, 28% beeswax	14
5	Brushed clean and coated with 6% palm oil, 6% pistacia resin, 88% beeswax	14

Following the embalming treatments, the post‐mummification samples were placed for 6 weeks in a food dehydrator set to 35°C. Each piece of skin and muscle from each embalming treatment was then divided into five sub‐sections, and each piece of bone into 10 (5 to be demineralised with HCl, 5 to be demineralised with EDTA). One sub‐sample of each was then either solubilised (soft tissues) or demineralised and solubilised without any cleaning method as follows:

### 2.1 Solubilisation

Approximately 250 mg tissue was placed in 7.5 mL water (pH 3) for 48 h at 70°C. The samples were then filtered through an 8‐μm Ezee filter, with the filtrate frozen at −20°C, then freeze‐dried.

### 2.2 Demineralisation with HCl

Bone samples were placed in 7.5 mL 0.5 M HCl for 5 days at 4°C. The samples were then rinsed three times in distilled water, then solubilised as above.

### 2.3 Demineralisation with EDTA^26^

Bone samples were placed in 30 mL 0.5 M EDTA (pH 8.0) for 5 days at 4°C, with the EDTA solution changed daily. The samples were then rinsed 15 times in distilled water, then solubilised as above.

The four cleaning methods being tested were then applied to the remaining subsections. Two of these cleaning methods (chloroform/methanol and methanol/chloroform/water) have been previously reported in the literature for stable isotope analysis of mummified remains. Another (dichloromethane) is commonly used to extract resinous materials from mummified remains for analysis by gas chromatography/mass spectrometry (GC/MS).^27^ This was included to test the possibility of using a single sample for both stable isotope and resin analysis. The last, toluene, was included as a similar but less‐toxic alternative to the use of benzene as recommended by Aufderheide et al.^17^

### 2.4 Chloroform/methanol^19^

Samples were ultrasonicated in 10 mL distilled water for 1 h, then soaked in 2:1 chloroform/methanol for 2 × 48 h, with sonication for 1 h after each change of solvent. They were then washed in 3 × 10 mL distilled water, and demineralised or solubilised as above.

### 2.5 Toluene

Samples were ultrasonicated in 10 mL distilled water for 1 h, then soaked in toluene for 2 × 48 h, with sonication for 1 h after each change of solvent. They were then washed in 3 × 10 mL distilled water, and demineralised or solubilised as above.

### 2.6 Methanol/chloroform/water^18^

Samples were soaked in 30 mL 10:5:4 methanol/chloroform/water at room temperature for 3 × 30 min. The samples were then soaked in 30 mL chloroform for 2 × 30 min, and 30 mL methanol for 2 × 30 minutes at room temperature. They were then air dried on filter paper at room temperature, and demineralised or solubilised as above.

### 2.7 Dichloromethane

Samples were soaked in 10 mL dichloromethane for 2 × 48 h, with sonication for 1 h after each change of solvent. They were then washed in 3 × 10 mL water, and demineralised or solubilised as above.

In all, this resulted in 100 samples for isotopic analysis; four different tissues (skin, muscle, bone demineralised with HCl, and bone demineralised with EDTA), with five embalming treatments each (1 fresh tissue control, 1 post‐mummification control, and 3 different coatings; see Table 1), with each of those being prepared according to five different cleaning methods (1 with no cleaning, 4 with different solvent cleaning methods). This is summarised in Table 2, which also provides the coding system used to identify each sample.

**Table 2:** Sample matrix

Tissue	Preparation					Cleaning method
	1	2	3	4	5	
S	S1N	S2N	S3N	S4N	S5N	N
	S1CM	S2CM	S3CM	S4CM	S5CM	CM
	S1T	S2T	S3T	S4T	S5T	T
	S1MCW	S2MCW	S3MCW	S4MCW	S5MCW	MCW
	S1DCM	S2DCM	S3DCM	S4DCM	SDCM	DCM
M	M1N	M2N	M3N	M4N	M5N	N
	M1CM	M2CM	M3CM	M4CM	M5CM	CM
	M1T	M2T	M3T	M4T	M5T	T
	M1MCW	M2MCW	M3MCW	M4MCW	M5MCW	MCW
	M1DCM	M2DCM	M3DCM	M4DCM	M5DCM	DCM
BH	BH1N	BH2N	BH3N	BH4N	BH5N	N
	BH1CM	BH2CM	BH3CM	BH4CM	BH5CM	CM
	BH1T	BH2T	BH3T	BH4T	BH5T	T
	BH1MCW	BH2MCW	BH3MCW	BH4MCW	BH5MCW	MCW
	BH1DCM	BH2DCM	BH3DCM	BH4DCM	BH5DCM	DCM
BE	BE1N	BE2N	BE3N	BE4N	BE5N	N
	BE1CM	BE2CM	BE3CM	BE4CM	BE5CM	CM
	BE1T	BE2T	BE3T	BE4T	BE5T	T
	BE1MCW	BE2MCW	BE3MCW	BE4MCW	BE5MCW	MCW
	BE1DCM	BE2DCM	BE3DCM	BE4DCM	BE5DCM	DCM

Key– Tissue: S (skin), M (muscle), BH (bone ‐ HCl), BE (bone ‐ EDTA)

– Preparation: 1 (fresh control), 2 (mummified control), 3 (100% pine resin), 4 (37% pine resin, 35% palm oil, 28% beeswax), 5 (6% palm oil, 6% pistacia resin, 88% beeswax).

– Cleaning method: N (none), CM (chloroform/methanol), T (toluene), MCW (methanol/chloroform/water), DCM (dichloromethane)**\.**

### 2.8 Stable isotope analysis

Freeze‐dried tissue samples were weighed in duplicate into tin capsules. Measurement of carbon and nitrogen isotope ratios was by continuous flow isotope ratio mass spectrometry (CF‐IRMS). The instrumentation comprised an elemental analyser (Flash/EA) coupled to a Delta Plus XL isotope ratio mass spectrometer via a ConFlo III interface (all provided by Thermo Fisher Scientific, Bremen, Germany). δ^13^C and δ^15^N values are expressed using the delta notation (δ) in parts per thousand (‰) relative to the international standards (VPDB and AIR) using an in‐house M1360p reference material (commercially available gelatine from British Drug Houses, Poole, UK) which has an expected δ^13^C value of −20.32‰ (calibrated against IAEA‐CH‐7 and NBS22) and a δ^15^N value of 8.12‰ (calibrated against IAEA‐N‐1 and IAEA‐N‐2). A modern cow bone standard (SADCOW) was used as a secondary check. The 1σ reproducibility for mass spectrometry controls for the analyses (repeated measures of the in‐house laboratory standard gelatine material) were ± 0.18‰ for δ^15^N values and ± 0.13‰ for δ^13^C values.

## 3 RESULTS

For this pilot study, resources were limited to the use of stable isotope analysis. Chromatographic or spectroscopic methods that could identify residual embalming materials following cleaning were not available. As such, the efficacy of each cleaning method is judged according to two criteria. The first is the ratio of carbon to nitrogen in the gelatinised collagen under analysis. The commonly accepted range for C/N ratios is 2.9–3.6,^28^ with ratios outside this range indicating contamination. The second criterion is the comparison of stable carbon and nitrogen isotope ratios from samples subject to embalming treatments 2–5 with the fresh control samples (embalming treatment 1).

### 3.1 Embalming materials

Table 3 shows the results of the stable isotope analyses of the materials used in embalming treatments 3–5. Despite a limit of detection of 0.1% for nitrogen, no nitrogen content was detected in any of these samples. As such, across the embalming materials and other reagents used, the only source of exogenous nitrogen is the EDTA used in demineralising one set of bone samples.

**Table 3:** Results of the stable isotope analyses of the embalming materials used

Treatment	δ^13^C values VPDB ‰	% C
Embalming treatment 3	−27.5	75.7
Embalming treatment 4	−27.1	82.2
Embalming treatment 5	−28.2	81.7

### 3.2 Skin

Table 4 shows the results of the stable isotope analyses of the skin samples. The fresh control samples, i.e. embalming treatment 1, all show C/N ratios well within the acceptable range, indicating a lack of any significant lipid contamination. This includes sample S1N, which underwent no cleaning method, suggesting that the solubilisation step alone is capable of extracting clean gelatin from untreated skin without the need for cleaning. As skin lipids are generally insoluble in polar solvents such as water or hydrochloric acid, this may be due less to a cleaning effect *per se*, and more to gelatin being solubilised while the lipids remained solid and thus being removed on filtration. As the C/N ratio for S1N is similar to that of the cleaned stable isotope samples, this suggests that the elevated temperature of the solubilisation step, and the hydrolysis of triacylglycerols to free fatty acids, did not appreciably increase the solubility of skin lipids in aqueous solutions.

**Table 4:** Results of the stable isotope analyses of skin from the mummified pig leg

Identifier	%C	%N	At C/N	δ^13^C VPDB ‰	δ^15^N AIR ‰
S1N	42.5	15.0	3.3	−22.9	4.7
S1CM	43.2	15.5	3.3	−22.7	4.7
S1T	44.5	15.8	3.3	−22.8	4.6
S1MCW	41.2	15.0	3.2	−22.9	4.7
S1DCM	43.9	15.6	3.3	−22.9	4.7
S2N	48.0	12.0	4.7	−24.8	4.6
S2CM	38.8	13.9	3.3	−22.9	4.7
S2T	45.8	14.8	3.6	−23.2	4.7
S2MCW	43.5	15.3	3.3	−22.7	4.8
S2DCM	46.6	13.7	4.0	−23.9	4.8
S3N	48.2	7.0	9.1	−27.4	3.7
S3CM	44.3	16.1	3.2	−23.0	4.5
S3T	46.0	14.0	3.9	−24.1	4.6
S3MCW	43.9	15.6	3.3	−22.8	4.5
S3DCM	44.1	15.1	3.4	−24.7	4.4
S4N	50.1	7.9	7.4	−26.1	4.4
S4CM	42.5	14.9	3.3	−22.8	4.5
S4T	42.3	15.7	3.2	−24.0	4.4
S4MCW	42.6	15.3	3.2	−23.0	4.7
S4DCM	43.7	14.5	3.5	−23.4	4.6
S5N	47.8	10.3	5.4	−25.2	4.5
S5CM	42.4	15.1	3.3	−22.8	4.6
S5T	50.1	11.6	5.1	−24.8	4.5
S5MCW	43.3	15.4	3.3	−22.9	4.6
S5DCM	44.2	14.5	3.6	−23.4	4.6

Each of the embalming treatment 1 skin samples show very similar stable isotope ratios, further suggesting that all the cleaning methods used were equally effective. They also provide baseline ratios of −22.9‰ δ^13^C/+4.7‰ δ^15^N against which the other skin samples can be compared.

The results for the skin samples that underwent embalming treatment 2 are very different. Two of the cleaning methods, chloroform/methanol and methanol/chloroform/water, produced very similar isotope ratios to the baseline results, and with C/N ratios within the acceptable range. The sample cleaned with toluene has a C/N ratio at the very upper end of the acceptable range, with a baseline δ^15^N value but very slightly depleted in ^13^C. The samples cleaned using methods 1 and 5, however, have C/N ratios above the acceptable range, and are significantly depleted in ^13^C, but have unaffected δ^15^N values. As no exogenous materials were used in this embalming method other than natron, the most probable explanations for the differences seen between these samples and the fresh control samples is that the mummification process itself changes the tissue or its components in some way that makes the endogenous lipids more difficult to remove, or that natron residues on or in the sample are not adequately removed during cleaning.

Determining which of these is the cause, or the extent to which they each contribute, is impossible to conclude without further analysis. Chromatographic analysis would be invaluable in determining whether lipids remain in the samples following preparation and, if so, in demonstrating how they have been modified. Although the lipids of ancient mummies have been studied, the results of those studies may be misleading if applied to a modern mummy as ancient examples have thousands of years of further post‐depositional changes beyond those caused by the mummification process itself.

The inclusion of residual natron within these samples is certainly a possibility, either from encrustations that were not removed when the samples were brushed clean, or from natron that had been absorbed by the tissue itself. That the depletions in ^13^C are most evident in those samples cleaned using the methods with the least amount of rinsing and soaking in water is certainly supportive of natron being at least a contributory factor. Unfortunately, the natron itself was not subject to stable isotope analysis at the same time as the tissue samples and embalming materials, so we cannot be sure of its likely impact if not fully removed.

However, the results for the skin samples that underwent embalming treatment 3 offer further support for the suggestion that residual natron is present. The chloroform/methanol and methanol/chloroform/water cleaning methods again produce samples with C/N ratios and δ^13^C and δ^15^N values very similar to those of the baseline values obtained from the fresh controls. Toluene cleaning resulted in a sample with a C/N ratio above the acceptable range, and depleted in ^13^C, while dichloromethane results in an acceptable C/N ratio, but even greater depletion in ^13^C than was seen for sample S3T. The root cause of these results is again difficult to explain, particularly that for S3DCM where the C/N ratio does not suggest any contamination, and no material with exogenous nitrogen was introduced. The results for S3N are strongly suggestive of residual natron. The C/N ratio is far above the acceptable range (9.1), and the sample is very depleted in ^13^C. The δ^13^C value of −27.4‰ is only slightly higher than that of pure pine resin (−27.5‰). That the sample had a nitrogen content of 7%, whereas embalming material 3 contained no measurable nitrogen content, suggests that the depletion seen is due to another carbon‐based contaminant even more depleted in ^13^C than the embalming material. For this experimental procedure, the only plausible source of this contaminant is the natron, which would appear to be highly depleted in^13^C.

Sample S4N shows a similar but less extreme set of results. Embalming material 4 has a δ^13^C value of −27.1‰ (Table 3), and no detectable nitrogen. Sample S4N has a C/N ratio of 7.4, and a δ^13^C value of −26.1‰, which is again suggestive of the inclusion of an exogenous carbon source with a lower δ^13^C value than the embalming material. The implications for the contaminants present in sample S5N are, however, more equivocal as the C/N ratio is lower, and the δ^13^C value higher than in S3N or S4N. Samples from embalming treatments 4 and 5, prepared using the chloroform/methanol and methanol/chloroform/water cleaning methods, show very similar results to both their equivalents in embalming treatments 2 and 3, and the baseline values from the fresh tissue controls. Samples S4DCM and S5DCM show similar results to S2T, i.e. a C/N ratio at the very top of the acceptable range and a slight depletion in ^13^C. Sample S4T has an acceptable C/N ratio but is rather depleted in ^13^C over the baseline values for fresh tissue. As with S3DCM, there is no clear cause for this. Sample S5T produced a C/N ratio beyond the acceptable range and a significantly lower δ^13^C value than the baseline. Whether this is because of natron contamination, lipid contamination, or both is hard to say from the results available.

### 3.3 Muscle

The results of the stable isotope analyses of muscle samples are shown in Table 5. Unlike the skin samples there is considerable variation in the results seen for embalming treatment 1, i.e. the fresh controls. Those samples undergoing no cleaning method (M1N) and cleaning with toluene (M1T) produced C/N ratios beyond the acceptable range. As no exogenous materials were added to these samples this indicates both that some cleaning is required for muscle samples, and that toluene is not a sufficient solvent using the method applied here. This is somewhat surprising, as the triacylglycerols and sterols that make up the great majority of endogenous lipids in animals are generally very soluble in non‐polar solvents such as toluene. The sonication stages in the cleaning method also noticeably warmed the solutions, which would have further increased the solubility of the lipids.

**Table 5:** Results of the stable isotope analyses of muscle from the mummified pig leg

Identifier	%C	%N	At C/N	δ^13^C VPDB ‰	δ^15^N AIR ‰
M1N	39.3	12.4	3.7	−24.8	3.2
M1CM	41.2	14.4	3.4	−23.5	4.6
M1T	44.1	13.4	3.9	−24.3	4.5
M1MCW	40.6	13.5	3.5	−23.9	3.7
M1DCM	41.8	13.8	3.5	−24.1	4.3
M2N	41.6	10.7	4.7	−25.0	3.9
M2CM	36.6	11.7	3.7	−24.3	4.2
M2T	48.8	12.3	4.6	−25.2	4.0
M2MCW	42.5	12.6	4.0	−24.3	4.2
M2DCM	47.4	12.4	4.5	−25.3	4.0
M3N	43.0	8.5	5.9	−25.3	4.0
M3CM	39.3	12.7	3.6	−24.1	4.0
M3T	43.5	15.5	3.3	−25.0	4.1
M3MCW	28.4	9.1	3.7	−24.0	3.8
M3DCM	48.5	12.0	4.7	−25.4	4.0
M4N	40.2	7.4	6.4	−25.1	3.8
M4CM	36.6	11.2	3.8	−24.2	4.1
M4T	48.0	11.5	4.9	−25.3	3.9
M4MCW	43.0	12.9	3.9	−24.4	3.9
M4DCM	48.2	12.6	4.5	−25.4	3.9
M5N	44.1	14.0	3.7	−24.6	4.1
M5CM	35.7	11.1	3.8	−24.1	4.8
M5T	42.6	15.8	3.1	−25.1	4.1
M5MCW	42.6	13.0	3.8	−24.2	4.2
M5DCM	45.6	12.6	4.2	−25.0	4.5

The remaining three samples, those cleaned with chloroform/methanol, with methanol/chloroform/water, and with dichloromethane, all had C/N ratios within the acceptable range. Their isotope ratios varied substantially, however, as can be seen in Table 5. With no additional data to inform which of these represents a more accurate baseline result than the others, a simple mean of the results has been used to provide this baseline, with the results from the other embalming treatments interpreted accordingly. As such, the muscle baseline values are −23.8‰ for δ^13^C values and +4.2‰ for δ^15^N values.

Nearly all the other muscle samples produced C/N ratios above the acceptable range, indicating contamination of some form. Without further analysis, it is not possible to say for sure whether this is due to endogenous lipids, embalming materials, or residual natron. The extreme variation seen in some of the skin samples is not seen for muscle. If that was indeed, as hypothesised, due to natron contamination, this may be because only the skin was in direct contact with the natron. This will certainly have prevented clumps of natron from adhering to the muscle tissue and being carried forward to analysis. The samples that underwent no cleaning method or that were cleaned in dichloromethane are not notably more depleted in ^13^C than those that had been cleaned with other methods, as was seen in skin. This suggests that natron is not a major source of contamination, as the lack of washing in water in the skin samples was associated with much lower δ^13^C values. Whether the contamination seen is due to endogenous lipids or exogenous embalming materials is not clear, and further analysis would be required to establish this.

Only two mummified samples had acceptable C/N ratios: M3T and M5T. These produced similar stable isotope ratios, as can be seen in Table 5. Although the nitrogen results are similar to the mean baseline identified from the control samples, the carbon isotope ratios are lower, at around −25‰ compared with just under −24‰ for the controls. Even with the variation seen in the control samples, the mummified samples appear to be quite depleted in ^13^C. Interestingly, the only samples that provided results similar to the calculated baseline results were those cleaned with chloroform/methanol and with methanol/chloroform/water. This is true across all the embalming treatments, other than sample M5CM, which appears enriched in ^15^N. Although this could be taken as suggesting that these cleaning methods are more reliable than the others, the combination of high C/N ratios for these samples and the lack of a clear baseline value against which to judge the results means that this is far from conclusive.

A possible explanation for the heterogeneity of the muscle isotope ratios observed is linked to tissue turnover rates. Muscle turns over on average every 40–60 days,^29^ quicker than dermal collagen, which turns over approximately every 2–4 months.^30^ It is thus possible that the shorter turnover time could result in differences in the muscle samples taken in terms of the period of formation that they represent. The increased consistency of isotope ratios seen in the skin and bone samples may be a result of the increased chance of selecting material that represents equivalent periods of formation.

### 3.4 Bone

The results of the analysis of bone samples demineralised with HCl are shown in Table 6. The control samples, from embalming treatment 1, all have acceptable C/N ratios and show very similar stable isotope results. From this, and weighting confidence slightly more towards those samples with a more rigorous cleaning method than BH1N, baseline values are taken as −23.4‰ δ^13^C and +4.3‰ for δ^15^N.

**Table 6:** Results of the stable isotope analyses of bone from the mummified pig leg, demineralised using HCl

Identifier	%C	%N	At C/N	δ^13^C VPDB ‰	δ^15^N AIR ‰
BH1N	44.2	15.8	3.3	−23.6	4.2
BH1CM	42.4	14.9	3.3	−23.4	4.4
BH1T	42.0	14.9	3.3	−23.4	4.3
BH1MCW	41.3	14.5	3.3	−23.4	4.3
BH1DCM	41.7	14.7	3.3	−23.3	4.3
BH2N	42.5	13.5	3.7	−23.7	4.2
BH2CM	42.7	14.9	3.4	−23.1	4.2
BH2T	42.5	14.9	3.3	−23.2	4.0
BH2MCW	43.3	15.0	3.4	−23.2	4.1
BH2DCM	42.4	14.9	3.3	−23.2	4.1
BH3N	43.7	13.9	3.7	−23.6	4.2
BH3CM	42.0	14.5	3.4	−23.1	4.2
BH3T	43.0	14.9	3.4	−23.3	4.2
BH3MCW	54.3	18.3	3.5	−23.3	4.3
BH3DCM	42.6	14.6	3.4	−23.2	4.1
BH4N	46.5	12.6	4.3	−24.4	4.2
BH4CM	42.1	14.7	3.4	−23.2	4.2
BH4T	43.2	14.7	3.4	−23.1	4.4
BH4MCW	41.4	14.1	3.4	−23.4	4.1
BH4DCM	42.1	14.5	3.4	−23.4	4.3
BH5N	43.1	14.3	3.5	−23.7	4.1
BH5CM	42.8	15.0	3.3	−23.4	4.2
BH5T	42.8	14.9	3.4	−23.4	4.2
BH5MCW	43.3	15.0	3.4	−23.3	4.2
BH5DCM	42.7	14.7	3.4	−23.2	4.2

Other than BH2N, which has a C/N ratio outside the acceptable range, the samples from embalming treatment 2 produced consistent results within the group. They also showed consistent, but very minor, enrichment in ^13^C and depletion in ^15^N, both around 0.2‰. The slight contamination and subsequent depletion in ^13^C seen in BH2N are most plausibly due to endogenous lipids, as the bone was not in direct contact with natron, and no other exogenous materials were added in this embalming treatment.

All the HCl‐demineralised samples which underwent no cleaning method showed similar stable isotope results, other than BH4N which had a C/N ratio much further beyond the acceptable range than the other samples. Why this particular sample proved resistant to cleaning is not clear, but if the embalming material is the cause, it is possible that palm oil is the most difficult to remove component as the results for embalming treatments 3 and 5 show that pine resin and beeswax can be substantially removed by the combination of demineralisation and solubilisation.

Each of the samples that were cleaned showed very similar results, with C/N ratios within the acceptable range, and carbon and nitrogen isotope ratios very similar to the baseline results. The apparent increased reliability of cleaning in these samples over the skin and muscle samples may be due to the inclusion of an additional stage in the form of demineralisation.

The stable isotope analysis results for the bone samples demineralised with EDTA are shown in Table 7. Unlike the samples demineralised with HCl, the embalming treatment 1 samples show some variation, particularly BE1N. This sample has a C/N ratio at the very top of, but still within, the acceptable range, and is notably depleted in ^13^C in comparison with both other BE1 samples. Averaging across the embalming treatment 1 samples gives baseline results of −24.6‰ for δ^13^C values and +4.0‰ for δ^15^N values.

**Table 7:** Results of the stable isotope analyses of bone from the mummified pig leg, demineralised using EDTA

Identifier	%C	%N	At C/N	δ^13^C VPDB ‰	δ^15^N AIR ‰
BE1N	39.8	13.0	3.6	−26.1	3.8
BE1CM	40.7	14.1	3.4	−24.2	4.1
BE1T	37.5	12.5	3.5	−24.6	4.0
BE1MCW	39.8	13.6	3.4	−24.3	3.9
BE1DCM	42.6	14.8	3.4	−24.0	4.3
BE2N	38.3	12.5	3.6	−25.0	3.8
BE2CM	43.1	15.0	3.4	−23.9	4.0
BE2T	43.7	14.9	3.4	−23.6	4.1
BE2MCW	41.7	14.2	3.4	−23.6	4.1
BE2DCM	44.2	15.2	3.4	−23.3	4.2
BE3N	40.2	10.2	4.6	−26.1	4.0
BE3CM	42.8	14.7	3.4	−23.4	4.1
BE3T	38.4	12.7	3.5	−24.5	3.9
BE3MCW	40.8	13.3	3.6	−24.1	4.0
BE3DCM	40.8	13.8	3.5	−23.7	4.0
BE4N	41.1	12.4	3.9	−24.8	4.1
BE4CM	41.3	14.4	3.4	−23.6	4.1
BE4T	42.1	14.5	3.4	−24.6	3.9
BE4MCW	39.9	13.1	3.6	−24.2	4.3
BE4DCM	41.2	14.0	3.4	−24.2	3.9
BE5N	42.1	13.5	3.7	−25.3	4.0
BE5CM	38.7	12.8	3.5	−24.7	3.8
BE5T	37.7	12.4	3.6	−25.2	3.5
BE5MCW	40.8	14.1	3.4	−24.6	3.8
BE5DCM	44.0	15.9	3.2	−23.5	3.9

The samples from other embalming treatments show the same general trends. Uncleaned samples had higher C/N ratios, generally above the acceptable range, and lower δ^13^C values than the baseline. The cleaned samples all have acceptable C/N ratios, but appear somewhat depleted in^13^C compared with the bone samples demineralised in HCl.

Insufficient demineralisation of bone is one possible cause for this variation, but apatite is enriched in ^13^C over the collagen fraction.^31^ Lipids are usually ^13^C‐depleted relative to collagen,^32^ as are the embalming materials used herein; so these in isolation or combination are the more probable cause. This is borne out by the generally higher δ^13^C values obtained for the samples in embalming treatment 2. The extent of contamination must be quite low, however, because the C/N ratios are within the acceptable range.

The extensive washing step at the end of the EDTA demineralisation stage makes it unlikely that residual EDTA contamination is the cause of this variation. Rather, the slightly higher, although still acceptable, C/N ratios of EDTA‐demineralised samples over the HCl‐demineralised samples suggests that there may be some lipid contamination.

### 3.5 Evaluation of cleaning methods

As the C/N ratios obtained for the majority of muscle samples are outside the acceptable range for stable isotope analysis, few if any conclusions regarding the efficacy of the cleaning methods tested herein can be drawn from the results for those samples. Further work is required to identify the nature of the contamination, which in turn would help to identify improvements to the cleaning methods for this tissue type.

The results for skin and bone samples do show some clear trends. In general, cleaning methods using a combination of polar and non‐polar solvents rather than a single solvent are more effective at removing both endogenous lipids and embalming materials. Being naturally derived materials, the embalming materials used in this study (and in ancient Egypt) would comprise a wide range of different substances. Some are similar to those found in the bodies themselves, such as palm oil, which is primarily made up of triacylglycerols,^33^ whereas others are very different. Beeswax, for example, includes a variety of esters, diesters and hydroxyesters.^34^ Resins contain large quantities of terpenes, with pine resin having large quantities of mono‐ and di‐terpenes,^35^ and pistacia resin being mainly triterpenes.^36^ The range of solubilities displayed is substantial. Wax esters, triacylglycerols and some terpenes will be generally more soluble in non‐polar solvents such as toluene or chloroform, for example, whereas other terpenes are more soluble in polar solvents. Fatty acids are generally insoluble in water at lower pH when they are fully protonated, whereas at higher pH they may be somewhat soluble.

As the single‐solvent cleaning methods used herein are based on non‐polar solvents, there is certainly a risk that some contaminants will not be removed. A combination of polar and non‐polar solvents allows for a wider range of substances to be removed, especially when the samples are repeatedly washed with water to remove residual natron. The apparent improvement in cleaning seen in those bone samples demineralised with HCl over those demineralised with EDTA may be due to the hydrolysis of esters and triacylglycerols to more polar molecules that are either more soluble in a particular solvent, or are soluble in a wider range of solvents, facilitating their removal. The EDTA solution used would not have allowed for the same level of hydrolysis in these substances, which may help to explain the greater variance in results seen in EDTA‐demineralised bone.

## 4 CONCLUSIONS

The results from this model show that stable isotope ratios can be significantly affected by mummification and the application of resins, waxes and oils. If the substances responsible for this effect are not removed, the results obtained could lead to the collection of misleading data. The experimental model presented here shows that tissue type, demineralisation method and cleaning method can also affect the data obtained.

Bone, when demineralised with hydrochloric acid, produces the most consistent results, probably because the demineralisation stage allows for improved removal of natron residues, and may increase the solubility of embalming materials and endogenous lipids. Greater variance is seen in bone samples demineralised with EDTA, suggesting that this demineralisation method is less effective at providing an initial cleaning stage than HCl demineralisation. Skin also produces generally consistent results when cleaned with a mixture of polar and non‐polar solvents. The single‐solvent methods tested are not, however, able to reliably remove embalming mixtures or natron residues.

Muscle, however, could not produce reliable results in this experimental model. Significant variation is seen across the results obtained for this sample, with possible causes including lipid contamination, ineffective removal of embalming materials or natron residues and poor preservation. The pretreatments used are not consistently successful in removing these contaminants, making it impossible to identify a reliable method for cleaning this tissue. As it can be difficult to distinguish between ancient mummified soft tissues without histological analysis, the refractory nature of muscle in comparison with skin may be particularly problematic.

In summary, skin and bone appear to represent the most reliable tissues for stable isotope analysis of mummified remains. Demineralisation with HCl for bone and pretreatment with a mixture of polar and non‐polar solvents produce accurate data from samples prepared using all the methods presented herein. However, muscle and bone that have been demineralised with EDTA have not consistently produced accurate data.

## References

[1] Lamb AL . Stable isotope analysis of soft tissues from mummified human remains. Environ Archaeol. 2016;21(3):271‐284.

[2] Iacumin P , Bocherens H , Mariotti A , Longinelli A . An isostopic palaeoenvironmental study of human skeletal remains from the Nile Valley. Paleo3. 1996;126(1–2):15‐30.

[3] Thompson AH , Richards MP , Shortland A , Zakrzewski SR . Isotopic palaeodiet studies of ancient Egyptian fauna and humans. J Archaeol Sci. 2005;32:451‐463.

[4] Touzeau A , Amiot R , Blichert‐Toft J , et al. Diet of ancient Egyptians inferred from stable isotope systematics. J Archaeol Sci. 2014;46(1):114‐124.

[5] White CD , Schwarz HP . Temporal trends in stable isotopes for Nubian mummy tissues. Am J Phys Anthropol. 1994;93:165‐187. 8147434 10.1002/ajpa.1330930203

[6] Basha WA , Chamberlain AT , Zaki ME , Kandeel WA , Fares NH . Diet reconstruction through stable isotope analysis of ancient mummified soft tissues from Kulubnarti (Sudanese Nubia). J Archaeol Sci. 2016;5:71‐79.

[7] Ikram S , Dodson A . The Mummy in Ancient Egypt. Cairo: The American University in Cairo Press; 1998.

[8] Metcalfe R . The Tutankhamouse experiment – Investigating tissue changes during mummification In: Gashe V, Finch J, eds. Current Research in Egyptology 2008. Bolton: Rutherford Press; 2008:81‐88.

[9] Lucas A , Harris JR . Ancient Egyptian Materials and Industries. London: Edward Arnold; 1962.

[10] Smith GE , Dawson WR . *Egyptian Mummies.* London: G. Allen & Unwin; 1924.

[11] Nissenbaum A , Buckley S . Dead Sea asphalt in ancient Egyptian mummies – Why? Archaeometry. 2013;55(3):563‐568.

[12] Proefke ML , Rinehart KL . Analysis of an Egyptian mummy resin by mass spectrometry. J Am Soc Mass Spectrom. 1992;3(5):582‐589. 24234502 10.1016/1044-0305(92)85036-J

[13] Nissenbaum A . Molecular archaeology: Organic geochemistry of Egyptian mummies. J Archaeol Sci. 1992;19:1‐6.

[14] Buckley SA , Evershed RP . Organic chemistry of embalming agents in Pharaonic and Graeco‐Roman mummies. Nature. 2001;413(6858):837‐841. 11677605 10.1038/35101588

[15] Jones J , Higham TFG , Oldfield R , O'Connor TP , Buckley SA . Evidence for prehistoric origins of Egyptian mummification in late Neolithic burials. PLoS ONE. 2014;9(8):1‐13. 10.1371/journal.pone.0103608 PMC4132097 25118605

[16] Cockitt JA , David AR . The radiocarbon dating of ancient Egyptian mummies and their associated artefacts: Implications for Egyptology In: Cannata M, ed. Current Research in Egyptology 2006: Proceedings of the Seventh Annual Symposium. Oxford: Oxbow Books; 2007:43‐53.

[17] Aufderheide AC , Nissenbaum A , Cartmell L . Radiocarbon date recovery from bitumen‐containing Egyptian embalming resins. JSSEA. 2004;31:87‐96.

[18] White CD , Longstaffe FJ , Law KR . Seasonal stability and variation in diet as reflected in human mummy tissues from the Kharga Oasis and the Nile Valley. Palaeo3. 1999;147:209‐222.

[19] Finucane BC . Mummies, maize, and manure: Multi‐tissue stable isotope analysis of late prehistoric human remains from the Ayacucho Valley. Perú J Archeol Sci. 2007;34:2115‐2124.

[20] Knudson KJ , Aufderheide AE , Buikstra JE . Seasonality and paleodiet in the Chiribaya polity of southern Peru. J Archaeol Sci. 2007;34(3):451‐462.

[21] Dawson WR . Contributions to the history of mummification. Proc R Soc Med. 1927;20(6):832‐854. 19985765 10.1177/003591572702000631 PMC2100864

[22] Arriaza BT . Beyond Death: The Chinchorro Mummies of Ancient Chile. Washington: Smithsonian Institution Press; 1995.

[23] Aufderheide AC . The Scientific Study of Mummies. Cambridge: Cambridge University Press; 2003.

[24] Garner R . Experimental mummification In: David AR, ed. The Manchester Museum Mummy Project. Manchester: Manchester Museum Press; 1979:19‐24.

[25] Zimmerman MR , Brier B , Wade RS . Brief communication: Twentieth‐century replication of an Egyptian mummy: Implications for paleopathology. Am J Phys Anthropol. 1998;107:417‐420. 9859878 10.1002/(SICI)1096-8644(199812)107:4<417::AID-AJPA4>3.0.CO;2-B

[26] Tuross N , Fogel ML , Hare PE . Variability in the preservation of the isotopic composition of collagen from fossil bone. Geochim Cosmochim Acta. 1988;52(4):929‐935.

[27] McCreesh NC , Gize AP , David AR . Ancient Egyptian hair gel: New insight into ancient Egyptian mummification procedures through chemical analysis. J Archaeol Sci. 2011;38(12):3432‐3434.

[28] DeNiro MJ . Postmortem preservation and alteration of in vivo bone collagen isotope ratios in relation to palaeodietary reconstruction. Nature. 1985;317(6040):806‐809.

[29] Moore DR , Phillips SM , Babraj JA , Smith K. Rennie MJ. Myofibrillar and collagen protein synthesis in human skeletal muscle in young men after maximal shortening and lengthening contractions. Am J Physiol – Endocrinol Metab 2005;288: 1153–1159. 10.1152/ajpendo.00387.2004 15572656

[30] Babraj JA , Cuthbertson DJ , Smith K , et al. Collagen synthesis in human musculoskeletal tissues and skin. Am J Physiol – Endocrinol Metab. 2005;289:864‐869. 10.1152/ajpendo.00243.2005 15972270

[31] Lee‐Thorpe JA , Sealy JC , van der Merwe NJ . Stable carbon isotope ratio differences between bone collagen and bone apatite, and their relationship to diet. J Archaeol Sci. 1989;16(6):585‐599.

[32] DeNiro MJ , Epstein S . Mechanism of carbon isotope fractionation associated with lipid synthesis. Science. 1977;197(4300):261‐263. 327543 10.1126/science.327543

[33] Sambanthamurthi R , Sundram K , Tan Y‐A . Chemistry and biochemistry of palm oil. Prog Lipid Res. 2000;39(6):507‐558. 11106812 10.1016/s0163-7827(00)00015-1

[34] Garnier N , Cecile C‐O , Rolando C , Regert M . Characterization of archaeological beeswax by electron ionization and electrospray ionization mass spectrometry. Anal Chem. 2002;74(19):4868‐4877. 12380806 10.1021/ac025637a

[35] Ludwiczuk A , Skalick‐Wozniak K , Georgiev MI . Terpenoids In: Badal S, Delgoda R, eds. Pharmacognosy: Fundamentals, Applications and Strategies. London: Academic Press; 2017:233‐266.

[36] Stern B , Heron C , Corr L , Serpico M , Bourriau J . Compositional variations in aged and heated pistacia resin found in late bronze age Canaanite amphorae and bowls from Amarna, Egypt. Archaeometry. 2003;45(3):457‐469.

